# Unfolding the collective functional potential of a synergistic multispecies community through genotypic and phenotypic analyses

**DOI:** 10.1016/j.bioflm.2025.100290

**Published:** 2025-05-24

**Authors:** Dana Ronin, Mads Frederik Hansen, Maximilian Lukas Flaig, Morten Kam Dahl Dueholm, Anders Ogechi Hostrup Daugberg, Joseph Nesme, Witold Kot, Mette Burmølle

**Affiliations:** a Section of Microbiology, Department of Biology, University of Copenhagen, Universitetsparken 15, 2100, Copenhagen Ø, Denmark; b Center for Microbial Communities, Department of Chemistry and Bioscience, Aalborg University, Fredrik Bajers Vej 7H, Aalborg, 9220, Denmark; c Section for Microbial Ecology and Biotechnology, Department of Plant and Environmental Sciences, University of Copenhagen, Thorvaldsensvej 40, 1871, Frederiksberg, Denmark

**Keywords:** Full genome sequencing, Social interactions, Microbial ecology, Microbial genetics, Synergism, Biofilm formation, Bioremediation

## Abstract

By studying model multispecies biofilm systems, we can further our knowledge regarding why some properties emerge solely in a multispecies setting. In this study, the model system under investigation is composed of four bacterial species: *Paenibacillus amylolyticus*, *Microbacterium oxydans*, *Stenotrophomonas rhizophila* and *Stenotrophomonas maltophilia*. This community was isolated from soil and has previously shown synergistic biofilm formation capabilities in vitro, along with other intrinsic properties, some of which could lead to potential industrial and agricultural applications. In this study, we conducted the first complete genome assemblies for these four strains and performed a manually curated annotation of the genomes to identify genomic features that could guide the selection of relevant phenotypic assays. In all four strains, we identified genes linked to interspecies communication, biofilm formation, secondary metabolite production, antibiotic resistance, enzymatic activity and metabolism of toxic xenobiotics. With metabolism being the largest gene function category identified, we then conducted growth assays on various carbon sources and relevant polysaccharides. This revealed interesting emergent behaviors - regarding growth and enzymatic activity - in the four-species community which were not seen in the monocultures. Overall, analysis of the complete genomes of this model community uncovered gene functions which could play a role in the previously observed community intrinsic properties, as well as provided insight to the positive social interactions observed in vitro.

## 1 Introduction

Bacterial communities in nature are often composed of a diverse pool of species retained within a structured extracellular matrix; this structure is called a biofilm. Within biofilms, numerous interactions take place - cells compete for space and nutrients while also sharing metabolites and benefitting from the presence of one another [1,2]. The structural dimension of the biofilm matrix allows for environmental and physiological heterogeneity, compartmentalization, and long-term coexistence [3,4]. Over time, coexistence can enable the evolution of a cooperative system, where community members engage in social activities with fitness benefits [[5], [6], [7]] or drive resource specialization [8]. During coexistence, microorganisms interact and modulate behavior of one another [9,10]. Recognizing the importance of community-intrinsic properties [11], there is a need to shift focus from monospecies to multispecies model systems [56].

We previously described a community of four species isolated from leaf litter, that exhibited a high-level of biofilm interdependency and community-intrinsic properties. The community is comprised of *Paenibacillus amylolyticus* DR949, *Microbacterium oxydans* DR951, *Stenotrophomonas rhizophila* DR952 and, what, until now, was referred to as *Xanthomonas retroflexus* DR953 [12]. Based on 16S rRNA gene identity and full genome phylogenetic placement, *X. retroflexu*s was now reclassified as *Stenotrophomonas maltophilia* DR953. Past studies showed that when these four species were combined in vitro, a 300 % increase in biofilm biomass was observed compared to when they were in isolation [12,13]. This specific community has other synergistic emergent properties, such as protection from protozoan grazing [14]. The synergy of this community has proved to be of commercial value, as these four species combined can also form biofilms on roots to increase drought tolerance in plants [15] and enhance degradation of waste products i.e. keratin [16]. So far, the underlying mechanisms of this synergy have been investigated via transcriptomics [17], proteomics [18] and metabolomics [19]. These approaches generated valuable insights but were limited to the specific conditions under which the experiments were conducted. Additionally, the heterogeneity caused by the spatial organization and gradients in a biofilm leads to marked differences in metabolic activity and activated pathways in cells positioned differently in the biofilm. Bulk -omics analyses are averages of such profiles, which do not conclusively elucidate the specific interactions driving gene expression in subpopulations.

To better understand the building blocks which make up our model four-species community, we conducted an in-depth bioinformatic analysis of the genomes, described annotations related to social interactions, and discussed how these genomic features might impact the specific functions of this community in a broader context. Some features identified were then analyzed with phenotypic assays including antibiotic resistance and carbon utilization profiles, as well as enzyme activity quantification. All in all, this is the first time that the complete and circularized genomes of this community have been annotated and analyzed to this extent, as well as linked to relevant phenotypic assays.

We identified genes in all four of our strains that encode interspecies density sensing, suggesting the potential for interspecies crosstalk; other genes associated with biofilm formation were also identified in all four strains, such as two-component systems, membrane transport, and flagella. Social genes were identified in all four of our strains, with *P. amylolyticus* encoding for the most. *P. amylolyticus* also had the greatest number of secondary metabolite biosynthetic gene clusters, having 16, while the other three strains each had two gene clusters. All four strains had the presence of KEGG pathways associated with the degradation of xenobiotic compounds, which included industrial chemicals and environmental pollutants. With metabolism being the largest category for the annotated genes for each of our community members, we tested the growth of our community in both mono- and four-species conditions on 96 different substrates using BIOLOG plates. We identified 20 substrates which enabled enhanced growth in the four-species community compared to monocultures, suggesting some level of cross-feeding. In addition, we identified that only certain combinations of two- or three-species allowed for cellulose and curdlan degradation. Identifying emergent traits in a model synthetic community is highly relevant for microbial ecology and biotechnological applications, helping us elucidate natural phenomena in ecosystems and teach us how to functionalize bacterial communities (i.e. biofertilizer, drought control, bioremediation). There is a plethora of things to be uncovered in the interactions occurring in this community, and here, we elucidate some of them through genomic analysis and subsequent phenotypic testing.

## 2 Results & discussion

### 2.1 Genome description and phylogeny

The four strains, *P. amylolyticus*, *M. oxydans*, *S. rhizophila*, and *S. maltophilia* (or as previously known, *X. retroflexus*), were originally isolated from decaying maize leaves placed in topsoil from Tåstrup Experimental Farms, Denmark [20]. The four isolates were sequenced with the Nanopore long-read platform supplemented with short-read Illumina sequencing for full genome data with high resolution. Following the complete genome assembly of the four genomes, we annotated the open reading frames (ORFs) and characterized the four isolates. *P. amylolyticus* had the largest genome of the four, more than 1.5x larger than that of *S. maltophilia*, which had the second largest genome. Corresponding to its relatively large genome, *P. amylolyticus* also encoded the most coding sequences (CDS) and tRNA counts (Table 1). *P. amylolyticus* was previously identified as a keystone species of this community, promoting plant growth, but only in the presence of the three other community members [21]; the size of the genome could be related to arising community functions. Interestingly, no plasmids were identified in any of the isolates.

**Table 1:** Characteristics of *Paenibacillus amylolyticus* DR949, *Microbacterium oxydans* DR951, *Stenotrophomonas rhizophila* DR952, and *Stenotrophomonas maltophilia* DR953. All genomes were assembled as complete circular genomes.

Strain	Organism	Genbank Accession	Average coverage (x): Illumina reads	Average coverage (x): Nanopore reads	Genome Size (bp)	G + C content (%)	rRNAs counts	tRNAs counts	CDS (coding sequences) counts
DR949	*Paenibacillus amylolyticus*	CP118896	148	144	7,329,239	45.68	26	103	6,922
DR951	*Microbacterium oxydans*	CP118897	310	213	3,994,899	68.60	6	46	3,833
DR952	*Stenotrophomonas rhizophila*	CP118898	235	125	4,227,346	66.77	10	68	3,757
DR953	*Stenotrophomonas maltophilia*	CP118899	262	149	4,680,266	66.25	13	70	4,383

**Fig. 1:**
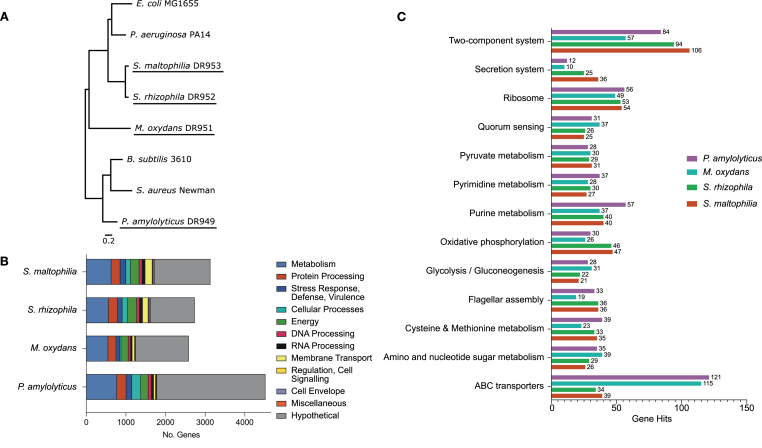
**General genomic descriptors. A**) Phylogenetic tree of the four species of interest (underlined), with well-studied model organisms *Pseudomonas aeruginosa*, *Escherichia coli*, *Staphylococcus aureus* and *Bacillus subtilis* as references. The tree is based on comparison of 1000 genes from each strain using the Mafft alignment of the BV-BRC bacterial phylogenetic tree tool [[25], [26]]. The bootstrap values were all 100. The genomes for the non-consortium strains were accessed either through BV-BRC database or through NCBI. **B**) The number of annotated genes for *Paenibacillus amylolyticus*, *Microbacterium oxydans*, *Stenotrophomonas rhizophila*, and *Stenotrophomonas maltophilia* classified by PATRIC into 11 systems. **C)** Distribution of top functional annotations based on the KEGG database. Cutoff value was set to categories where one or more species had 30 gene hits or more. The y-axis is the KEGG annotation pathways, and the x-axis is the number of corresponding genes.

When analyzing the genetic distance between the isolates, it was not surprising that the two isolates of the *Stenotrophomonas* genus were close in the phylogenetic tree (Fig. 1A). On the other hand, *M. oxydans,* of the Actinobacteria phylum, is relatively isolated in the phylogenetic tree. *P. amylolyticus* has closer proximity to the two Gram-positive model organisms (*Bacillus subtilis* and *Staphylococcus aureus*) introduced as references in this tree; these three species are Gram-positives and belong to the Bacillota/Firmicutes phylum, which is generally recognized as GC-poor [22,23]. This is also reflected in our community, where *P. amylolyticus* has the lowest GC content of the four bacteria (45.68 %, Table 1).

To classify the function of the genes, all CDS were annotated using PGAP [24], categorized into eleven default categories suggested by the BV-BRC software [25,26], as well as categorized by a KEGG pathway analysis. For all four isolates, many genes were categorized as involved in ‘metabolism’ or ‘protein processing’ (Fig. 1B). Many CDS had unknown functions; *P. amylolyticus* encoded 2,737 (40 %) genes with hypothetical functions, while *M. oxydans*, *S. rhizophila*, and *S. maltophilia* had 1,342 (35 %), 1,116 (30 %), and 1,409 (32 %), respectively. Despite *P. amylolyticus'* large genome, the number of genes that are categorized is only marginally higher than for the two *Stenotrophomonas* species (Fig. 1B), most likely due to *P. amylolyticus* having almost double the hypothetical proteins compared to the other three species. Notably, the two Gram-positive bacteria, *P. amylolyticus* and *M. oxydans*, have fewer genes in the ‘membrane transport’ category than the two *Stenotrophomonas* species. This could potentially be a consequence of the additional outer membrane, and presence of the periplasm in Gram-negative bacteria, which requires more complex transportation systems [27,28]. However, conducting a functional annotation analysis of the four genomes using the KEGG database revealed that *P. amylolyticus* and *M. oxydans* have substantially more genes involved in ABC (ATP-binding cassette) transporter pathways than the two Gram-negatives (Fig. 1C). ABC transporters couple ATP hydrolysis to translocate various substances, and hence enable uptake of nutrients or secretion of toxins in an ATP-dependent fashion [29]. This suggests that our community's Gram-positive members have a less diverse catalogue of membrane transporter types and rely on active transportation of substrates. In line with that, the two *Stenotrophomonas* species have 2-3-fold more genes involved in secretion system pathways than the Gram-positives (Fig. 1C); therefore, these two species either 1) have a broader range of transporter systems, or 2) require more genes to pass a substrate beyond the outer membrane.

All four isolates contain genes involved in two-components systems (Fig. 1C), which allow for recognizing specific environmental cues and responding accordingly, including regulation of biofilm formation [30,31]. Noticeably, *M. oxydans* only encodes 57 genes involved in two-component system pathways, which is relatively few compared to the three other species of the community (*S. maltophilia* is the top scorer with 106 genes (Fig. 1C)). On the other hand, *M. oxydans* encodes for most genes related to quorum sensing (QS). QS involves production, secretion and detection of signal molecules that reflect the bacterial density, and thus, enables a collective control of gene expression and synchronized group behavior according to cell density. Commonly, signal molecules are referred to as autoinducers and differ between bacterial species. Gram-negative bacteria produce different homoserine lactones as signal molecules and Gram-positive bacteria produce oligopeptides as autoinducer signals [32]. Autoinducer-2 (AI-2) is a universal signal molecule produced by both Gram-negative and Gram-positive bacteria, enabling interspecies density sensing. All AI-2 producers encode a *luxS* gene homolog essential for AI-2 synthesis [32,33]. When searching for similar genes of the Pfam family of *luxS* in the genomes of the four strains, *P. amylolyticus* had three matches, while *M. oxydans*, *S. rhizophila*, and *S. maltophilia* all had one match (Suppl. Table 1), thus indicating the potential for crosstalk and interspecies density sensing in the community.

Although a complete genome greatly improves the quality and reliability of a genomic analysis, there are still gaps in our knowledge greatly driven by the number of hypothetical annotations. Given this four species community is composed of non-model organisms, it makes it even harder to identify these unknowns. Additionally, is it difficult to draw conclusions about community functions from genotypic descriptors. Therefore, it is critical to supplement such genomic analysis with phenotypic assays to generate more concrete conclusions on the findings.

### 2.2 Matrix genes for united biofilm formation

Motivated by the high level of biofilm synergy between these four isolates [12,13,34], we started searching for genes associated with biofilm formation. As discussed above, we have identified numerous genes for two-component systems, membrane transport and quorum sensing (QS). These genes play significant roles in both direct and indirect interspecies interactions, including biofilm regulation. The AI-2 mediated crosstalk can, for example, promote and alter biofilm formation [35,36]. Next, genes involved in flagellar assembly were identified in all four species (Fig. 1C). While the primary function of the flagella is motility, it is also of great importance in terms of initial attachment to substrates [37]; the flagella function as anchors for various matrix components, such as cellulose and amyloids [38,39]. Hence, the flagella could potentially be vital in the interaction of matrix components of the different species and ultimately facilitate an important part of the biofilm synergy. This is supported by a recent study on these four strains, which showed a differential abundance of flagellin proteins in the mono- and multispecies biofilm matrices [40]. The genes encoding for Fap amyloids [41] were identified in both *S. rhizophila* and *S. maltophilia*.

**Fig. 2:**
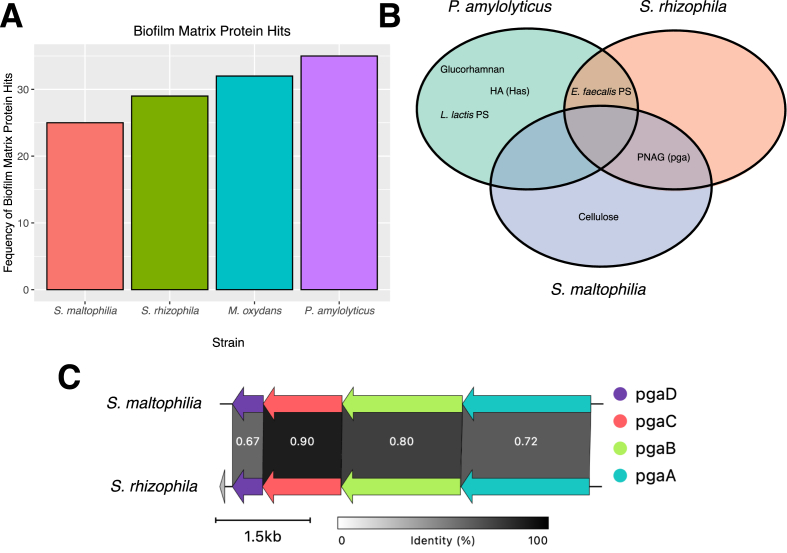
**Biofilm matrix genes. A)** The frequency of hits which matched the NCBI database for ‘biofilm matrix’ protein hits. The protein matches between the strains and the database were then filtered to only include matches with a percent identity of 70 % or above. We refer to Suppl. Table 2 for the comprehensive list of hits. B) Venn diagram with hits from the exopolysaccharide analysis. *Paenibacillus amylolyticus* encoded for hyaluronic acid (Has), glucorhaman, *Lactococcus lactis* polysaccharides and *Enterococcus faecalis* polysaccharides. *Stenotrophomonas rhizophila* encoded for *E. faecalis* polysaccharides and PNAG. *Stenotrophomonas maltophilia* encoded for PNAG and cellulose. C) Gene cluster comparison of PNAG shared between the two *Stenotrophomonas* species. Greyscale intensity indicates amino acid similarity between genes, with the exact similarity indicated by the number in white text.

While there are no previous reports on the direct correlation between the number of genes coding for matrix proteins and biofilm formation, past studies have demonstrated how various matrix components allow for better protection of cells in a biofilm [42,43]. Biofilm matrix components have functions such as providing a protective environment, retaining exoenzymes, protecting from enzymatic degradation, stabilizing the matrix and transporting components [34]. The number of genes encoding biofilm matrix proteins was estimated in each of the four isolates (Fig. 2A). A protein database based on a NCBI “biofilm matrix” keyword search was the reference for identifying those genes in our isolates. Due to its relatively large genome, it was not surprising that *P. amylolyticus* had the greatest number of matrix proteins. Nonetheless, it was surprising that *S. maltophilia* had the lowest number of hits for matrix proteins, as this species is often the best single-species biofilm former of these four [[13], [34]]. In accordance, it was also a surprise to find that *M. oxydans* had the second greatest number of matrix protein gene hits (Fig. 2A), given that *M. oxydans* cannot form biofilms on its own, nor does it compose much of the biofilm in the four-species community in certain setups [44,45]. Nevertheless, biofilms consist of other biopolymers besides proteins. For example, the biofilm phenotype of *Vibrio cholerae* changes dramatically when deleting genes involved in synthesis of vibrio exopolysaccharides (*vps* genes) [46]. In a search for other biofilm related genes, we identified known gene clusters involved in exopolysaccharide synthesis using the Dueholm et al. [47] pipeline. Here, we got no matches in the genome of *M. oxydans* but identified two operons in both *S. maltophilia* and *S. rhizophila*, and four operons in *P. amylolyticus* (Fig. 2B). Specifically, *P. amylolyticus* encoded the potential to produce hyaluronic acid (Has), glucorhamnan, and two variants of polysaccharides that resemble those previously identified in *Lactococcus lactis* and *Enterococcus faecalis*. These polysaccharides could potentially have functions other than biofilm formation, given polysaccharides are common components of Gram-positive bacteria cell walls [48,49]. Interestingly, the *E. faecalis* PS operon was also identified in the Gram-negative *S. rhizophila*. A closer inspection of the synteny of the PS operons identified in *P. amylolyticus* and *S. rhizophila*, compared to the *E. faecalis* query, revealed their differences. Each operon had multiple genes not present in the query; both lacked a gene involved in product modification, as well as multiple genes involved in polymerization (Suppl. Fig. 1). Thus, the PS operons identified in our genomes are likely producing an alternative variant of the polysaccharide. On the contrary, the PNAG operon in *S. rhizophila* and *S. maltophilia* was similar to the PNAG query, with comparison of the operons from both species showing a high amino acid similarity between the four genes of relevance (*pgaABCD*) (Fig. 2C). PNAG is a known matrix component that is important for biofilm formation in various Gram-positive and -negative bacteria [[50], [51], [52], [53]]. Another point to emphasize is the presence of the exopolysaccharide cellulose solely in *S. maltophilia.* Cellulose is a known important structural component in biofilms, providing a type of hydrogel with strong water adsorption properties [54]. The presence of the cellulose exopolysaccharide in *S. maltophilia* could serve as an explanation as to why this community member often forms substaintial biofilms as a monoculture [13].

### 2.3 Identification of genes with social function

**Fig. 3:**
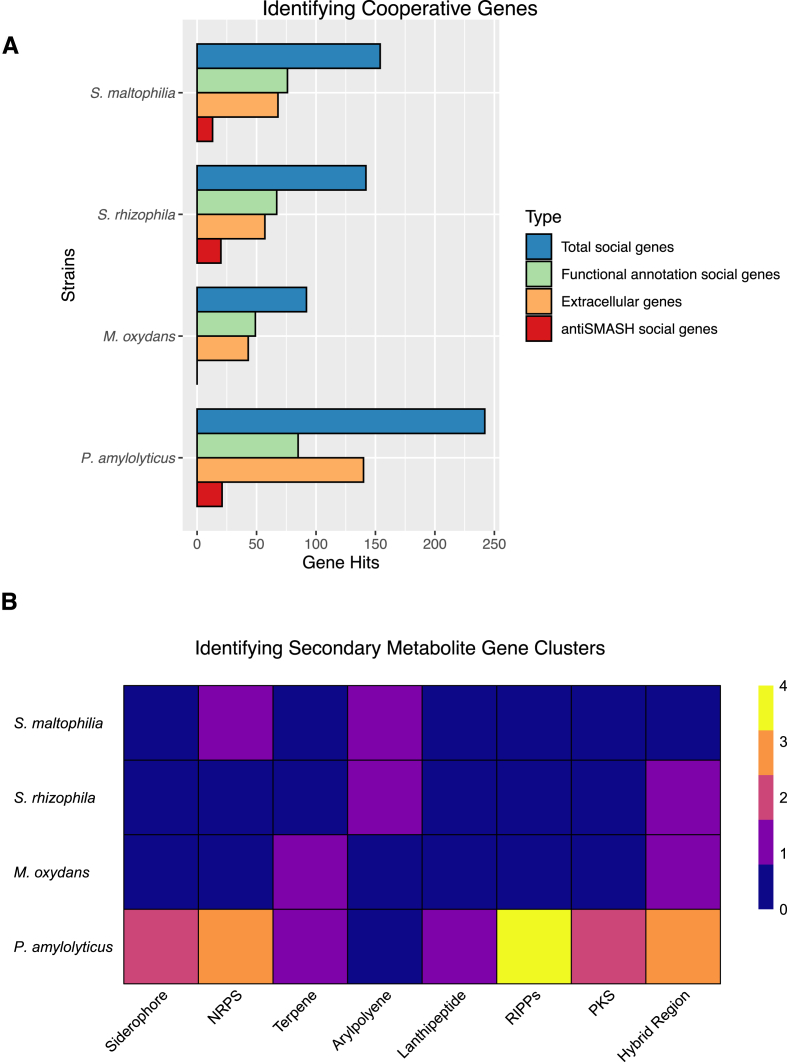
**Social genes. A)** Cooperative genes were identified in our four genomes using SOCfinder. The cooperative genes focused on here are those identified as having a functional annotation, encoding for extracellular proteins, and the antiSMASH genes which encode secondary metabolites. The total counts of cooperative genes are also shown to account for any overlap. **B)** The standalone antiSMASH pipeline was used to identify secondary metabolite biosynthetic gene clusters in *Paenibacillus amylolyticus*, *Microbacterium oxydans*, *Stenotrophomonas rhizophila*, and *Stenotrophomonas maltophilia* (see full list of genes in Suppl. Table 3).

In addition to potential sharing of matrix components and interspecies signaling, a community can also benefit from other social interactions. The SOCfinder pipeline [55] searches genomes to identify genes that control social traits (‘social genes’). The software identifies genes encoding secreted molecules, cooperative functions and secondary metabolites, all of which are expected to play a role in social interactions. The tool counts several genes that make up one ‘trait’ only once. When we applied the software to the four species in this community, *P. amylolyticus* had the greatest number of total social genes relative to the other three strains (Fig. 3A). *M. oxydans* had the lowest total number of social genes. Previous studies have demonstrated cooperative interactions between *S. maltophilia* (previously *X. retroflexus*) and *P. amylolyticus*, showing their interactions on multiple levels in the four-species community [17,18,45,57,58]. Other studies have shown that *M. oxydans* cooperatively interacted with *S. maltophilia* to stabilize the rest of the four-species community [59], and was essential for the more advantageous spatial positioning of *S. rhizophila* and *S. maltophilia* at the top of the biofilm [44].

The SOCfinder software did not identify any cooperative genes involved in synthesis of secondary metabolites for *M. oxydans* (Fig. 3A). A closer inspection using the standalone antiSMASH [60] to characterize the clusters for secondary metabolite synthesis revealed that *M. oxydans* encoded for two putative clusters for production of secondary metabolites (hybrid region and terpene). This discrepancy might be because: 1) SOCfinder excludes the loosely defined hybrid regions 2) terpene does not fulfill the classification for being cooperative or 3) terpene synthase genes are often silent [61]. Looking at the antiSMASH results, *P. amylolyticus* showed a markedly greater number of clusters (16) compared to the other three community members, each of which had solely two synthetic gene clusters (Fig. 3B). From a social perspective, the fact that *P. amylolyticus* encodes a potential siderophore is of interest. Microorganisms that produce siderophores are iron scavengers; the production of these chelating molecules can function as public goods by enabling producers and non-producers to benefit from acquiring this essential trace element [62,63]. This observation suggests that the other community members may benefit from the presence of *P. amylolyticus* under certain conditions. On the other hand, *P. amylolyticus* may be scavenging the limited iron for itself, which would limit the essential resource for surrounding organisms; this behavior would then effectively enhance competition and potentially lead to an antagonistic relationship with other organisms [64]. In the two *Stenotrophomonas* spp., they differed in one of the gene cluster categories. Both species have the potential to produce arylpolyene; however, *S. maltophilia* encodes a non-ribosomal peptide synthesis (NRPS) cluster, while *S. rhizophila* encodes a hybrid region.

**Fig. 4:**
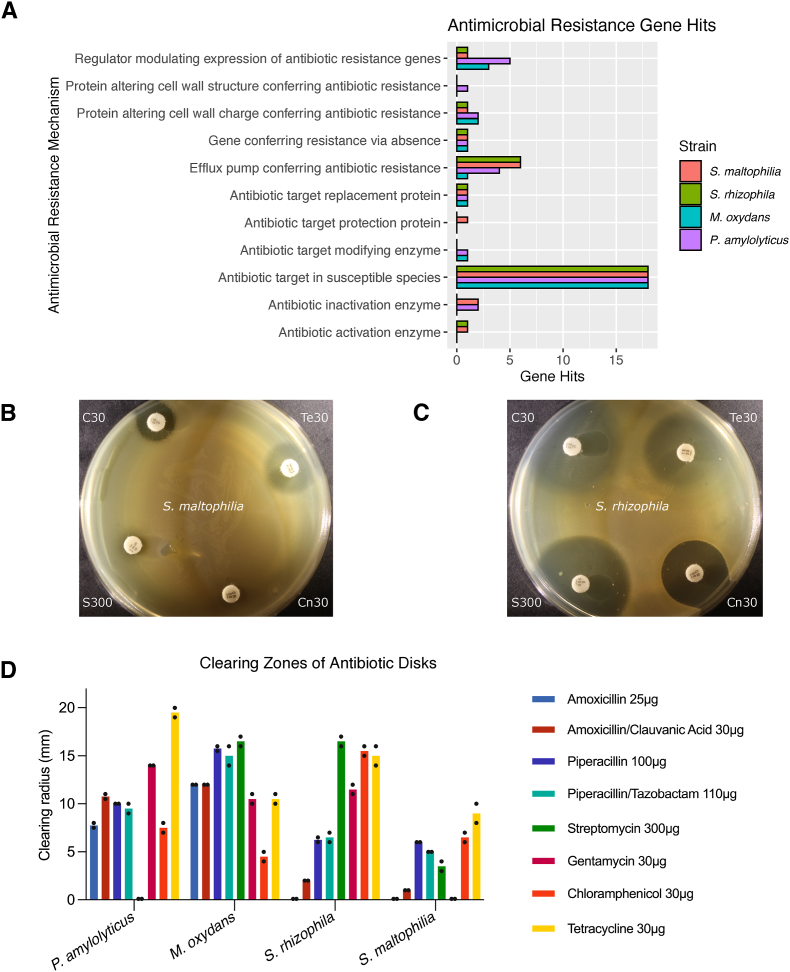
**Antibiotic resistance. A)** Genes encoding antibiotic resistance determinants, and the respective antimicrobial resistance mechanisms, were identified using the antibiotic resistance k-mer identification method in the PATRIC software [[25], [26]]. The frequency of the gene hits for each category were quantified. The full list of antibiotic resistance genes for each strain can be found in Suppl. Table 4. **B)** The susceptibility of the four strains to antibiotics on a surface was tested using Oxoid^TM^ antibiotic disks. Pictured are four of the antibiotics tested, chloramphenicol 30 μg/mL (Cm30), tetracycline 30 μg/mL (Te30), streptomycin 300 μg/mL (S300), and chloramphenicol 30 μg/mL (CN30), when placed on top of a lawn of bacteria. Here, we see that *Stenotrophomonas maltophilia* generally grew well in close distance to these four drugs tested, while **C)***Stenotrophomonas rhizophila*, in contrast, showed high sensitivity to all drugs tested in this assay. **D)** The antibiotic resistance profiles of the four strains were quantified as clearing zone measurements (mm) around antibiotic disks added on top of a lawn of bacteria inoculated on top agar.

Other genes that can function as public goods are those encoding enzymes that degrade antibiotics, such as those encoding secreted β-lactamase enzymes [65]. Using the PATRIC software [25,26], we found that all four strains encoded antibiotic resistance genes, including efflux pumps, with the two *Stenotrophomonas* spp. encoding most efflux components (Fig. 4A). Of interest, *P. amylolyticus* and *S. maltophilia* also encoded antibiotic inactivation enzymes (Fig. 4A). Overall, the genetic analysis did not indicate large differences in the number of genes involved in antibiotic resistance (Fig. 4A and Suppl. Table 4). The number of genes, however, does not necessarily explain the phenotypic profile. For example, efflux pumps can vary in promiscuity [66,67]. Examining the specific PATRIC-identified genes revealed that the two *Stenotrophomonas* spp. encode the same genes annotated as efflux pumps, including EmrAB-TolC, a well-known efflux pump that causes increased broad-range resistance when tested in *E. coli* [68]. *S. maltophilia* uniquely encoded the aminoglycoside phosphotransferase (APH(3’)), while *P. amylolyticus* encoded VanXY and an O-acetyltransferase of the CatA15/A16 family, which confer resistance to vancomycin [69] and chloramphenicol, respectively (Suppl. Table 4). O-acetyltransferases of the CatA15/A16 family have, however, frequently been observed in *Bacillus* spp. without yielding phenotypic resistance [70]. Finally, we identified Tet42 in *M. oxydans*, a putative efflux pump that shares similarity to the tetracycline resistance determinant TetA(Z) [71].

To determine if these genes confer phenotypic resistance, growth was quantified in proximity to discs impregnated with different antibiotics. All strains exhibited a clearing zone around chloramphenicol (Fig. 4D), supporting that the presence of the CatA15/A16 family O-acetyltrasferase often does not result in chloramphenicol resistance. Next, we tested two aminoglycosides, and found that *S. maltophilia* was completely resistant to gentamycin and had a very small clearing zone around streptomycin (Fig. 4B + D), indicating that the APH(3′) aminoglycoside phosphotransferase confers resistance to aminoglycosides [72]. Given the high similarity of genomic resistance profiles between *S. rhizophila* and *S. maltophilia*, as well as the high sensitivity of *S. rhizophila* to gentamycin and streptomycin, it is likely that the APH(3’) gene specifically confers the aminoglycoside resistance to *S. maltophilia* (Fig. 4C + D). Interestingly, *P. amylolyticus* was completely unaffected by the presence of streptomycin; to further verify this finding, we grew colonies of *P. amylolyticus* on TSA supplemented with up to 5000 μg/mL streptomycin and did not observe a reduction in generation time compared to when grown in TSB supplemented with 400 μg/mL streptomycin (data not shown). *P. amylolyticus* encodes FosB, which confers resistance to the phosphonic acid fosfomycin [73], RlmA(II) which confers resistance to macrolides [74], the aforementioned VanXY, and two efflux systems, BceAB and ykkCD. The latter, ykkCD, has especially been found to associate with a multidrug-resistant phenotype [75]; since streptomycin is neither a phosphonic acid, macrolide, or glycopeptide, we suspect that this efflux pump could be the main driver of the streptomycin resistance. Finally, all strains seemed sensitive to tetracycline, which disputes that the Tet42 encodes for a functional tetracycline efflux pump in *M. oxydans.*

Antimicrobial resistance gives a fitness advantage to microorganisms in the presence of that antibiotic; however, understanding the effects of antimicrobial resistance becomes a challenge at the community level, given that resistance in one species may be conferred to the other, sensitive community members. In fact, it has been recently shown that in a multispecies community, one species carrying a resistance plasmid managed to detoxify the environment for the other, sensitive species. The effect and the mechanism, however, varied between species, with some benefitting more from the production of β-lactamases than others [65]. In *S. rhizophila* and *S. maltophilia*, two of the social genes identified were categorized as β-lactamases (Suppl. Table 5). We also demonstrated the ability of these two strains to grow in presence of β-lactams (amoxicillin and piperacillin), both alone and in combination with their corresponding β-lactamase inhibitors (clavulanic acid and tazobactam). Both clavulanic acid and tazobactam function by ‘suicide inhibition’. They irreversibly bind to β-lactamase enzymes and form a stable acyl-enzyme complex. This complex mimics the intermediate formed from β-lactams, effectively inactivating β-lactamase enzymes [76,77]. We observed that the strains were resistant to amoxicillin, but the presence of clavulanic acid renders the strains sensitive (Fig. 4D). Pairing tazobactam with piperacillin did not inhibit resistance, indicating the *Stenotrophomonas* spp. produce β-lactamases not affected by the tazobactam, but sensitive to clavulanic acid. A similar trend has also been seen for a pOXA48 plasmid-carrying *E. coli* strain, encoding a β-lactamase not seemingly inhibited by tazobactam [65]. Using antibiotic discs on a bacterial lawn for these assays allowed us to measure the antibiotic resistance profile in surface-associated biofilm conditions, rather than in a planktonic culture.

### 2.4 Metabolism and niche expansion

**Fig. 5:**
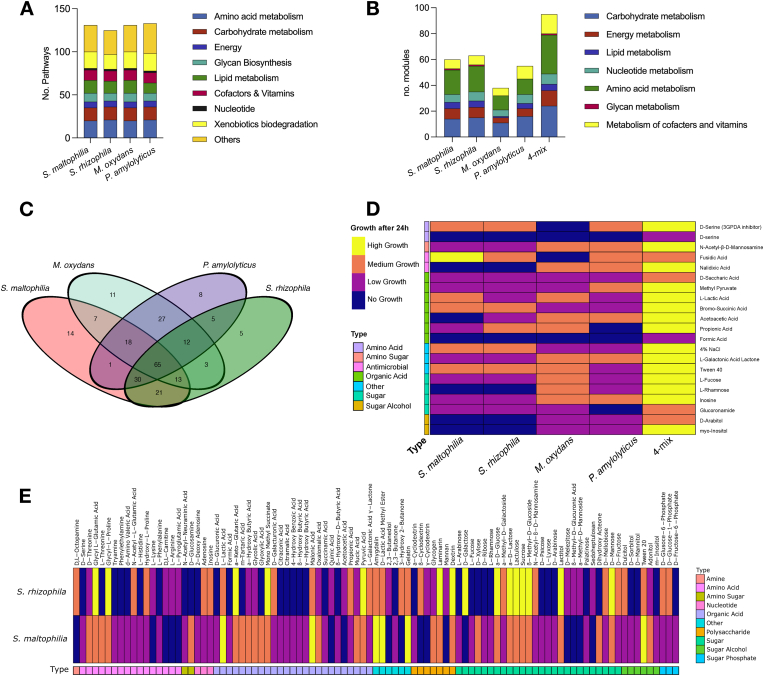
**Metabolism. A)** Depiction of number of metabolic pathways identified in the four strains categorized by KEGG category. **B)** Number of metabolic pathway modules identified in each strain, as well as the four-mix community. **C)** Venn Diagram depicting the total number of substrates from all three BIOLOG microplates that each strain can utilize for growth. Data of the substrates in the final three columns of the GENIII plate were excluded for the creation of this diagram, as they test for growth on antibiotics, different salt concentrations and pH levels. Specific substrates that enable growth for each species can be found in Suppl. Fig. 2ABC. **D)** Heatmap showing bacterial growth on various substrates from GenIII plates, which allows for Gram-positive and -negative species mixing. 570 nm endpoint absorbance values were recorded, and then categorized into no growth, low growth, medium growth, and high growth. Substrates shown here had higher growth for the four-mix community compared to any of the individual species. Fusidic acid is the sole substrate where *S. maltophilia* had higher growth compared to the four-mix community. The full data for all 96 substrates tested for the GenIII plate is shown in Suppl. Fig. 2A. **E)** Heatmap depicting the substrates from all three plates for which the two *Stenotrophomonas* species exhibited different growth patterns. Further differences between the two strains are highlighted in Suppl. Fig. 3.

Most of the annotated genes in the four strains were associated with metabolism (Fig. 1B). To decipher which processes these genes enabled, we used the KEGG mapper reconstruct tool [78] to identify full functional metabolic modules. Despite the large variation in genome size among the strains (Table 1), the number and distribution of pathways were quite similar (Fig. 5A). For the two *Stenotrophomonas* spp., a total of 129 metabolic pathways were identified, of which 123 were shared between them (full list in Suppl. Table 6). The differential pathway present in *S. rhizophila* was C21-Steroid hormone metabolism, whereas in *S. maltophilia*, brassinosteroid biosynthesis, aeatin biosynthesis, toluene and xylene degradation, and isoflavonoid biosynthesis were present. 132 metabolic pathways were identified in *P. amylolyticus,* and 131 pathways were identified in *M. oxydans* (Fig. 5A). Given these bacteria are of interest due to their emergent properties as a community, we also identified the total number of modules, while only counting reappearing modules once. Here, it was noteworthy that the categories for carbohydrate, energy and amino acid metabolism expanded compared to the single species analyses (Fig. 5B), indicating that species rely on different metabolisms and highlight a potential for cross-feeding. Noticeably, all four strains had a similar number of pathways associated with degradation of xenobiotic compounds (Fig. 5A), and there were several xenobiotics which all four strains had the genomic capacity to degrade. Other studies have identified that some strains of *Microbacterium* and *Stenotrophomonas* possess xenobiotic degradation capabilities [79]. Specifically, *S. rhizophila* and *S. maltophilia* have previously demonstrated both genomic and phenotypic capacities for the degradation of compounds used in pollutants and pesticides, as well as plant growth promotion abilities [80,81].

Xenobiotics, among other things, are often industrial chemicals and environmental pollutants. We identified pathways for the degradation of γ-hexachlorocyclohexane (also known as lindane), 1- and 2 methylnaphthalene, bisphenol A, benzoate, styrene, 1,4-dichlorobenzene, tetrachloroethene, caprolactam, trinitrotoluene, naphthalene, anthracene, ethylbenzene and geraniol in all four isolates (Table 2). Xenobiotics can have direct and indirect detrimental effects to humans, animals, plants and ecosystems [79]. Some studies showed that exposure to xenobiotic pollution changed the gut microbiome of humans, by actively selecting for xenobiotic degrading bacteria in the gut [82]. Degradation of xenobiotic pollutants by microbes is an environmentally sustainable approach for remediation of pollution of the environment, with a number of patents currently making use of this [79]. Whether our four-species community could effectively be used for the remediation of the listed xenobiotics in a natural setting remains to be tested.

**Table 2:** List of xenobiotic compounds for which biodegradation pathways were identified in all four of our strains. The Comparative Systems tool from BV-BRC was used for pathway identification.

Xenobiotic compound	Origin	Effects on human health	Effects on environment	Citation
γ-Hexachlorocyclohexane	Insecticide (agriculture, topical application to skin)	Skin irritation, dizziness, headaches, diarrhea, nausea, vomiting, convulsions, seizures and death	Persists in environment, toxic to animals, enters food chain by bioaccumulation in animals	[[83], [84], [85]]
1- and 2-Methylnaphthalene	Crude oil, byproducts of combustion	Decreased pain sensitivity; limited data on humans	Bioaccumulative; ubiquitous in environment but impact on ecosystems uncertain	[86]
Bisphenol A	Polycarbonate plastics (e.g., water bottles, baby bottles, epoxy resins)	Reduced fertility, eye damage, allergic skin reactions and respiratory irritation, hormone system disruption	Endocrine-disrupting effect on fish, amphibians and invertebrates; biodegradable in water	[87,88]
Benzoate	Food, cosmetic, and drug preservative	Low acute toxicity, may cause non-immunological contact reactions	Contamination of aquatic systems and organisms	[[89], [90], [91]]
Styrene	Production of plastics and rubber	Mucous membrane irritation, eye irritation, gastrointestinal effects; disruption of central nervous system	Mild toxicity to aquatic organisms; volatile	[139]
1,4-Dichlorobenzene	Fumigant (moth repellant), space deodorant (toilets and refuse)	Irritation to the eyes, skin, and throat, adverse effects on liver, skin, central nervous system	Low potential for ecological risk	[[92], [136]]
Tetrachloroethene	Dry-cleaning, degreaser, intermediate in chemical production	Irritation of the upper respiratory tract and eyes, kidney dysfunction, and neurological effects, likely carcinogenic	Common pollutant of aquatic systems, low chronic and acute toxicity to aquatic organisms	[93]
Caprolactam	Manufacture of synthetic fibers (nylon)	Irritation of eyes, nose, throat, and skin, headaches	Low potential for ecological risk	[135]
Trinitrotoluene	Explosive used in military shells, bombs, and grenades	Abnormal liver function, blood disorders (e.g., anemia)	Potential metabolization by plants	[137]
Naphthalene	Fossil fuels, production of chemical intermediates (e.g., phthalic anhydride)	Headache, nausea, vomiting, diarrhea, confusion, anemia, convulsions, coma, cataracts	Persists in environment but little to no bioaccumulation or ecotoxicity	[[94], [138]]
Ethylbenzene	Styrene production, solvent	Throat and eye irritation, chest constriction, neurological effects	Moderate ecotoxicity hazard	[95]
Geraniol	Perfumes, cosmetics, personal care products and pharmaceuticals	Little to no risk, allergic reaction in some individuals	Low potential for ecological risk	[96]
Anthracene	Fumes from burning organic materials such as coal, oil, tobacco, and wood	Skin and eye irritation, respiratory problems, potential carcinogenicity	Bioaccumulative and potential for long-term contamination, toxic to aquatic organisms	[97]

To phenotypically verify the metabolic capabilities identified on the genomic level, the utilization of different substrates was tested using BIOLOG PM1, PM2 and GenIII microplates. Each plate had 96 substrates; however, due to some overlap between the plates, the total carbon sources tested were 237 for the monocultures, and 94 for the four-species community. In line with the genomic analysis, indicating a similar number of complete pathways present in each strain (Fig. 5A), we found that each strain was able to grow on a similar number of substrates (*S. maltophilia* = 169, *S. rhizophila* = 154, *M. oxydans* = 156 and *P. amylolyticus* = 166, Fig. 5C). The large genome of *P. amylolyticus* could indicate that this species is a generalist that utilizes a broad range of substrates. However, this was not the case. *P. amylolyticus* was only able to uniquely utilize eight substrates, which was the second lowest among the strains (Fig. 5C). We speculate that *P. amylolyticus* may have incomplete metabolic pathways, requiring other species for metabolism of certain substrates.

It was previously shown that combining species enables the utilization of carbon sources otherwise inaccessible for strains in monoculture. Specifically, 16 species isolated from a watermelon rhizosphere were able to grow on glycyl-l-glutamic acid, d-glucosamic acid and α-keto butyric acid when grown together, while none of the 16 isolates were able to grow on these carbon sources alone [98]. Similarly, a separate study reported that a four-species community benefitted from each other's presence when grown in industrial metal working fluid containing biocides; this medium was only able to support the growth of one of the species as a monoculture [99]. While the PM1 and PM2 plate protocols varied depending on Gram classification of the bacteria, the GenIII microplates did not, making it possible to mix our four-species community and test if it showed similar abilities for niche expansion. To distinguish between growth ability on various substrates, the endpoint absorbance (570 nm) was recorded; these values were then classified into four categories (no growth, low growth, medium growth, and high growth) for ease of visualization and relevance. The combination of four species resulted in growth on two carbon sources (d-serine and formic acid) otherwise unavailable for the species when growing as monocultures (Fig. 5D). Further, we also identified 20 different substrates on which the four-species community showed higher growth, and thus improved substrate utilization capabilities, compared to any of the individual species alone, suggesting potential niche expansion for the four-species community (Fig. 5D). Finally, only a single substrate was identified on which a single species showed better substrate utilization in monoculture compared to the community (*S. maltophilia* on fusidic acid, Fig. 5D).

The metabolic analyses also highlighted the differences between the two *Stenotrophomonas* spp.: *S. maltophilia* was able to utilize more unique substrates than *S. rhizophila*. In fact, 40 of the 169 substrates *S*. *maltophilia* was able to utilize did not support growth of *S. rhizophila*, which instead was able to utilize 25 substrates unsupportive of *S. maltophilia* growth (Fig. 5C). When dissecting the differences between the two *Stenotrophomonas* species, there were 95 substrates on which they exhibited different substrate utilization capabilities; it was evident that especially amino acids, organic acids, and sugars were sources that supported growth differentially (Fig. 5E).

When inoculating the plates in standing culture conditions as per Sun et al. [100], we acknowledge that some of the cells may be in in the planktonic state; thus, the results observed could be an average of combined biofilm and planktonic cells, where different unknown proportions of each are present. However, in past studies of this community, we observed a high degree of synergy in these four species only in the biofilm setting [34]. While we have not tested substate utilization in a pure planktonic culture, we can assume that there would be less interactions between the cells, thus potentially showing fewer emergent properties.

**Fig. 6:**
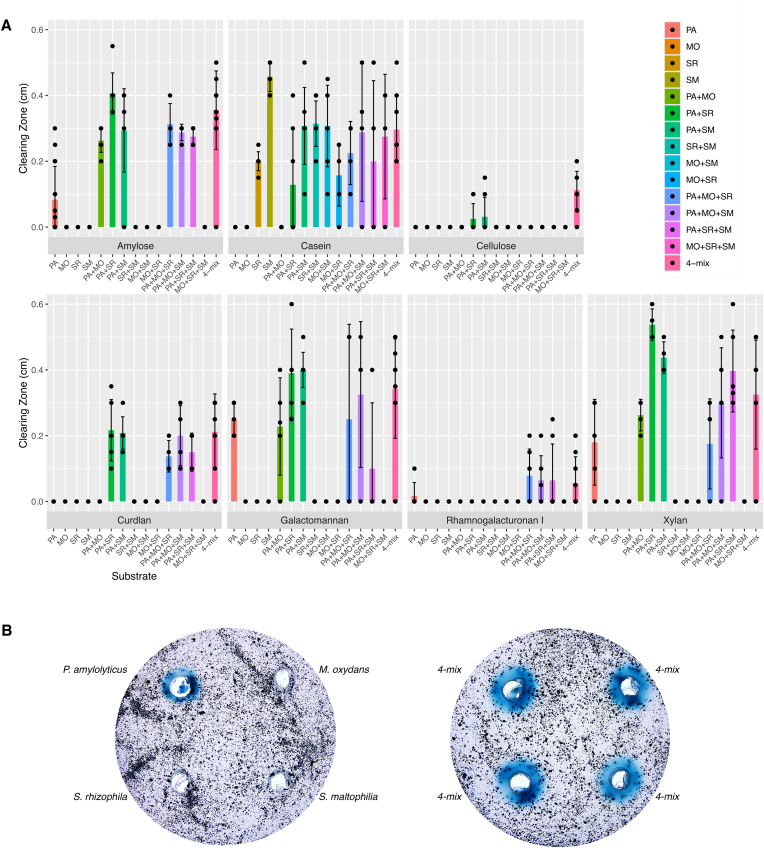
**Enzymatic capacities. A)** The enzymatic capacities of the *Paenibacillus amylolyticus* (PA), *Microbacterium oxydans* (MO), *Stenotrophomonas rhizophila* (SR), and *Stenotrophomonas maltophilia* (SM) were assessed in monocultures, as well as in four-mix combinations. The substrates tested were the azurine-crosslinked polysaccharides (AZCL): amylose, casein, cellulose, curdlan, galactomannan, rhamnogalacturonan I, and xylan. A minimum of two technical replicates were conducted for each substrate tested. **B)** For the substrates assessed which had enhanced enzymatic capacities in the four-mix combination, further two-mix and three-mix combinations were conducted to further understand the key player driving the degradation abilities. **C)** An image of the AZCL xylan plates pictured, with both monoculture inoculations and four-mix inoculations (confirmed with three biological replicates).

Based on the observed carbon source utilization profiles and given this community has been previously shown to enhance degradation of recalcitrant compounds [16], we decided to quantify and compare the enzymatic activity of the community with that of the monocultures. For this, we used seven azurine-cross-linked substrates (amylose, casein, curdlan, cellulose, xylan, galactomannan and rhamnogalacturonan I) which release a dye upon enzymatic cleavage. All (except casein) are of ecological relevance in context of where this community was isolated. Amylose is an important carbon source which many bacteria can use for energy [101]. The degradation of casein can be applied for bioremediation strategies, such as the cleanup of dairy waste. Curdlan is produced by other soil bacteria [102], while cellulose and xylan are abundant biopolymers in plant material, serving as decomposition substrates that support microbial growth. Galactomannan is commonly found in leguminous seeds, which means it may be found in the soil [103], and rhamnogalacturonan I is commonly found in the cell walls of plants [104]. Quantification of visible clearing zones after incubation revealed an intriguing pattern for many of the substrates; cellulose and curdlan were only digested in the multispecies communities (dual-, triple- and four-species combinations) (Fig. 6). Even given that *P. amylolyticus* is a known cellulose degrader [105,106], it was not able to degrade the substrate as a monospecies in this set up. Furthermore, *P. amylolyticus* was the sole strain able to degrade amylose, galactomannan and xylan as a monoculture; however, the presence of the other three bacteria elevated the degradation (Fig. 6). To pinpoint which community members were necessary for the increased community-intrinsic degradation, we quantified clearing zones in dual- and triple-species communities. Again, *P. amylolyticus* was essential for degradation of all compounds, except casein (Fig. 6), where *S. rhizophila* and *S. maltophilia* showed degradation abilities as monocultures (Fig. 6). Pairing either *Stenotrophomonas* spp. with *P. amylolyticus* induced greater enzymatic activity on xylan. Overall, the importance of community interactions was highlighted by degradation of cellulose and curdlan, where the presence of *P. amylolyticus* and a minimum one of the *Stenotrophomonas* spp. was necessary for enzymatic activity (Fig. 6).

Building on this finding, and to further understand the emergent cellulose and curdlan degradation occurring only in multispecies communities, we conducted a genomic search for the relevant gene markers. In our search for cellulases, the following keywords were used: cellulase, glucanase, glucosidase, glucanohydrolase, cellobiohydrolase, cellodextrinase, β-glucosidase, cellobiose phosphorylase, cellodextrin phosphorylase, cellobiose epimerase [107,108]. We identified different types of cellulases in each of our strains (Table 3). Interestingly, cellulases, like our bacterial community of interest, work synergistically together [108]. This could be one reason why we see an increased degradation of cellulose in the dual- and four-species combinations with *P. amylolyticus*. Also, there are certain cellulases that are known to have synergistic interactions between them: exo- with endo-cellulases, several exocellulases, β-glucosidases with other cellulases, and cellobiohydrolase with endoglucanases and β-glucosidases [108]. This could explain the potentially synergistic cellulase activity when *P. amylolyticus* is paired with other partners.

**Table 3:**
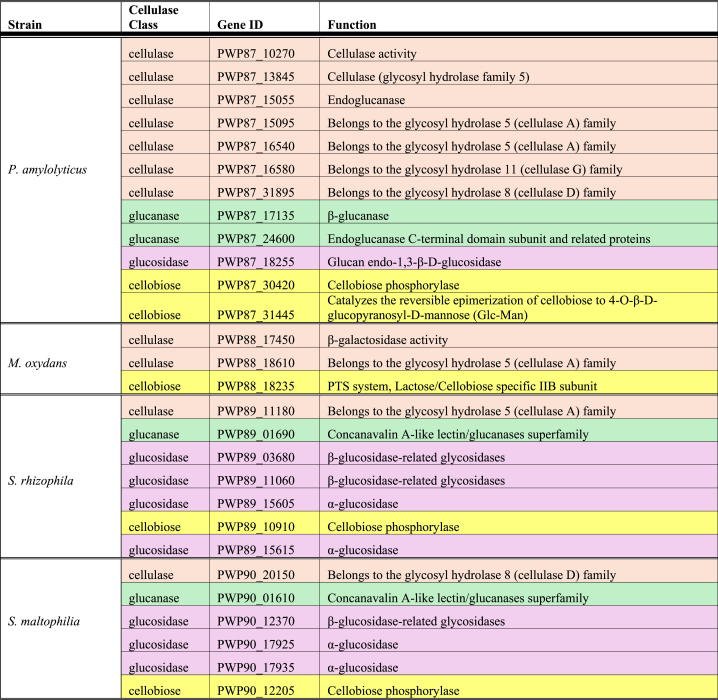
Genes identified relating to cellulose degradation. The relevant genes which were searched for were: cellulase, glucanase, glucosidase, glucanohydrolase, cellobiohydrolase, cellodextrinase, β-glucosidase, cellobiose phosphorylase, cellodextrin phosphorylase, cellobiose epimerase. Different cellulase classes are shown in the different colors.

For curdlan degradation, the main enzyme responsible is glucanase. We identified three genes for glucanase in *P. amylolyticus*, and one in both *S. rhizophila* and *S. maltophilia* (see Table 4). The specific enzyme responsible for curdlan degradation is β-glucanase, which only *P. amylolyticus* has in the genome. We speculate that the genes identified in *S. rhizophila* and *S. maltophilia* may also help to fully degrade curdlan, which *P. amylolyticus* is unable to do alone.

**Table 4:** Genes identified relating to curdlan degradation, with glucanase being the relevant gene searched for.

Strain	Gene ID	Function
*P. amylolyticus*	PWP87_15055	Endoglucanase
	PWP87_17135	β-glucanase
	PWP87_24600	Endoglucanase C-terminal domain subunit and related proteins
*S. rhizophila*	PWP89_01690	Concanavalin A-like lectin/glucanases superfamily
*S. maltophilia*	PWP90_01610	Concanavalin A-like lectin/glucanases superfamily

This enzymatic analysis highlights the necessity of pairing genotypic and phenotypic assays. While the AZCL assays gave us an insight into the community function, the identification of the relevant genes helps explain the mechanism occurring behind the catalysis.

## 3 Conclusion – ecological relevance & future applications

Past studies have experimentally assessed phenotypes resulting from specific interactions within this four-species consortium. Meanwhile in this study, we bioinformatically analyzed the genome of each community member on its own to potentially explain the community interactions in an ecological context. To uncover the mechanisms behind the in vitro interactions we observe in this community, we took a bottom-up approach to a model bacterial community usually studied through a macro lens. With this approach, we identified multiple genes involved in crosstalk, cross-feeding, antibiotic resistance, biofilm formation, metabolism, xenobiotic degradation, and enzymatic activity. This genome analysis approach helps pinpoint relevant functional assays and subsequently identify the mechanistic foundation of the observed community interactions.

Following the bioinformatic analysis, we identified and tested several relevant phenotypic assays, which truly highlighted the community-intrinsic potential of this community. Specifically, as a four-species community, the biofilm exhibited enhanced growth on various nutrient sources and collectively degraded compounds such as cellulose and curdlan – compounds that none of the species could break down individually.

In general, being in a multispecies community can give rise to a plethora of new, collective behaviors. Such behaviors include enhanced antibiotic tolerance, metabolic cooperation, resistance to protozoan grazing, protection against bacteriophages, and increased biomass [109]. This also can work in reverse, where a high stress, or toxic, environment can drive facilitation between the bacterial community members, allowing for improved survival as a multispecies community [99]. Other multispecies biofilm communities have applicable emergent properties, such as enhanced disease resistance in plants [110,111], improved surface colonization [112], and functioning as biofertilizers [113]. These observations emphasize the need to study multispecies communities. The emergent properties observed in multispecies communities have a plethora of industrial and environmental applications. One such application is bioremediation, or the use of microorganisms in the removal of pollutants without further generating toxic waste. Various studies have previously demonstrated the emergence of enhanced bioremediation behaviors in a multispecies community. In fact, the involvement of multiple species in bioremediation can allow for improved biodegradation through sequential biological reactions and metabolic cross-feeding [114]. In this study, we identified 13 pathways for xenobiotic biodegradation; testing the degradation of these compounds experimentally is critical for understanding the full degradation potential of our community. Another potential application is the use of this community in agriculture as a biocontrol; by experimentally confirming (or rejecting) several of the identified antibiotic resistance genes, we now have a better overview of a risk assessment for the implementation of this community in a less controlled, natural environment.

In this study, we demonstrated that genomic analysis can be a useful tool in predicting collective traits in a multispecies setting by effectively streamlining experimental processes. Nevertheless, while this kind of workflow allows for more directed phenotypic testing, it is still limited in throughput. Various relatively new tools have been developed to allow for the metabolic modeling of several bacterial species in varying levels of environmental complexity [115,116]. Such tools merge the use of genomic and experimental data to output key bacterial interactions based on complex and spatially structured environments.

## 4 Experimental procedures

### 4.1 Culture conditions

The four strains of focus, *P. amylolyticus*, *M. oxydans*, *S. rhizophila*, and *S. maltophilia* were cultured as previously described [21]. Briefly, these four strains were streaked from glycerol stocks onto tryptic soy agar (TSA) plates (3 % (W/V) TSB (Millipore), 1.5 % (W/V) agar). After a 48 h incubation at 24 °C, a colony was picked and inoculated in 5 mL of TSB at 250 rpm at 24 °C or 30 °C.

### 4.2 Sequencing, genome assembly and annotation

The four isolates were sequenced with the Nanopore long-read platform supplemented with short-read Illumina sequencing for full genome data with high resolution. The Illumina sequences for these four strains were available at the ENA repository (*P. amylolyticus*: PRJEB15262; *M. oxydans*: PRJEB15265; *S. rhizophila*: PRJEB15263; *S. maltophilia*: PRJEB18431). For Nanopore sequencing, the genomic DNA of *P. amylolyticus*, *M. oxydans*, *S. rhizophila*, and *S. maltophilia* was isolated with the DNeasy Ultra Clean Microbial Kit (Qiagen 1222450). The library was prepared with a rapid barcoding kit 96 (Nanopore SQK-RBK110.96) and sequenced as part of the PromethION flowcell FLO-PRO002 (based on R9.4.1 pores), following the manufacturer's instructions. For the *de novo* genome assembly, Nanopore reads were quality filtered with Filtlong v. 0.2.1 [117], adapter trimmed with Porechop v. 0.2.4 [118] and assembled with Flye v. 2.9.1 with the –nano raw software setting, the default mode for Oxford Nanopore technologies [119]. The Illumina reads were quality and adapter filtered with Fastp v. 0.23.2 [120]. Using a long-reads-first hybrid assembly approach, the Flye assembly was inputted into Bowtie2 v. 2.5.1 [121] to generate an index, allowing for the Illumina reads to be mapped onto the Flye assembly. Samtools v. 1.16.1 [122] was used to sort and index the output bam file. Pilon v. 1.24 [123] was used as a polishing step, to improve the accuracy of the draft genomes by fixing errors like single base differences, local misassemblies and block substitution events. Circulator v. 1.5.5 [124] was used with the –fixstart software setting to change the position of all circularized genomes to begin at DnaA. Qualimap v. 2.2.2d [125] was used with the –bamqc flag to output genome coverage of the Illumina reads mapped onto the Flye assembly. NCBI Prokaryotic Genome Annotation Pipeline (PGAP) [24,126] was used for genome annotation. The raw data and assemblies have been deposited in GenBank under the BioProject accession number PRJNA937621 (see Table 5). The PGAP annotations were also inputted into the EggNOG-Mapper tool [127] to obtain more detailed annotations (i.e. for hypothetical proteins).

**Table 5:** **Bacterial strains and reference genomes.** List of organism names and accession numbers for the bacterial strains used in this study.

Organism	Assembly Accession (NCBI)
*Paenibacillus amylolyticus* DR949	CP118896
*Microbacterium oxydans* DR951	CP118897
*Stenotrophomonas rhizophila* DR952	CP118898
*Stenothrophomonas maltophilia* DR953	CP118899
*Escherichia coli* MG1655	GCF_000801205.1
*Pseudomonas aeruginosa* PA14	GCA_000404265.1
*Bacillus subtilis* 3610	GCA_002055965.1
*Staphylococcus aureus* Newman	GCA_002310435.1

### 4.3 Functional clustering of annotations and identification of cooperative genes

Genome features and assembly details were obtained with the Comparative Genome Analysis tool from BV-BRC web resources [26]. This resource also outputted information about the antimicrobial resistance encoding genes, xenobiotic biodegradation pathways, as well as allowed us to construct a phylogenetic tree of relatedness including model strains as references (see Table 5). The KAAS tool [131] was utilized to categorize the KEGG pathways. The KEGG mapper reconstruct tool [78] was also used to identify full functional metabolic modules.

An NCBI search for identical protein groups using the keyword “biofilm matrix” was conducted to generate a database of 1800 biofilm matrix proteins. This database was then used as a reference to identify biofilm matrix proteins in our four strains. A threshold of 70 % identity match was set to filter the results. Hypothetical proteins were discarded from being considered as matches. For identification of genes involved in polysaccharide synthesis, we converted CDS of respective genomes into gff format and uploaded these to the Dueholm et al. [47] pipeline. The PNAG operons (+200bp flanking regions) of the two *Stenotrophomonas* spp. were uploaded to the CAGECAT platform [128] to perform and visualize a comparison on the amino acid level.

When searching for the *luxS* homolog in our four strains, the pfam entry PF02664 for S-Ribosylhomocysteinase (LuxS) was used as the reference. A database was created using this superfamily of proteins, which was then blasted against the four strains. A filter of 85 % was set to exclude irrelevant matches. The antiSMASH tool was used to identify secondary metabolite gene clusters in the genomes [60]. Cooperative genes in each of the four strains were identified using SOCfinder [55].

### 4.4 Characterization of metabolic and chemical properties with BIOLOG plates

With the use of carbon utilization assays PM1 (BIOLOG Cat #12111) and PM2 (BIOLOG Cat #12112) microplates, we tested the ability of the four strains to grow on various carbon sources. We based our protocol on a previous study [100]. A fixed concentration of bacteria was used, at approximately 1–4 x10^8^ CFU/mL corresponding to exponential cultures at OD_600_ = 0.2 for *S. rhizophila*, *S. maltophilia* and *M. oxydans* and OD_600_ = 0.6 for *P. amylolyticus* according to the OD_600_ to CFU/mL standard curve (Suppl. Fig. 4).

The following protocol was used to test the substrate usage of the Gram-negatives *S. rhizophila* and *S. maltophilia*, in PM1 and PM2 BIOLOG plates. A wash solution (4.5 mL sterile water mixed with 22.5 mL of 1.2X IF-0a) and a distribution solution (7 mL of sterile water, 37.5 mL of 1.2x IF-0a, and 0.54 mL dye mix A) were mixed. 5 mL of OD-adjusted cell cultures were centrifuged at 10,000 rcf for 3 min, after which the supernatant was removed and the pellet washed with washing solution. This was repeated twice. Each washed culture was then mixed with 20 mL of distribution solution. To mix, the tubes were inverted slowly eight times. 100 μL of each culture mixture was then added to the wells of PM1 and PM2 plates.

To test for substrate utilization of the Gram-positive bacteria, *P. amylolyticus* and *M. oxydans*, on PM1 and PM2 BIOLOG plates, the protocol was modified slightly. First, the 12X PM additive solution was mixed (30 mL sterile water, 10 mL 0.6 % tween80, 10 mL 0.6 % yeast extract, 30 mL of a solution containing 0.5 mM l-cystine at pH 8.5 and 1 mM 5′-UMP, 10 mL solution containing 3 mM l-arginine and 6 mM l-glutamate, 10 mL solution containing 240 mM MgCl_2_ and 120 mM CaCl_2_). Then, a wash solution (1.8 mL sterile water mixed with 9 mL of 1.2X IF-0a) and a distribution solution (40 mL of 1.2X IF-0a, 0.48 mL dye mix H and 4 mL 12X PM additive solution) were prepared. 2 mL of OD-adjusted cell cultures were centrifuged at 10,000 rcf for 3 min, after which the supernatant was removed and the pellet washed with washing solution. This was repeated twice. 1.76 mL of the washed cultures were mixed with 22.24 mL of distribution solution. 100 μL of each culture mixture was added to the PM1 and PM2 plates.

The protocol for testing substrate utilization with GENIII plates (BIOLOG Cat # 1030) did not vary depending on Gram classification. 10 mL of OD-adjusted cultures were centrifuged at 10,000 rcf for 3 min, after which the supernatants were discarded and 5 mL of IF-A solution was added. This was repeated twice. The final pellets were resuspended in 10 mL of IF-A solution. 100 μl of the culture mixture was added to the GENIII plate.

All BIOLOG microplates were incubated at 24 °C for 24 h before growth as 570 nm endpoint absorbance was measured in a microplate reader (Biotek Synergy H1). Two biological replicates were done. The data for each bacterial strain were normalized against their respective negative controls and then used to create heatmaps [129]. The values were categorized in the following manner: values less than or equal to 0 were labelled as “no growth”. Terciles for the remaining data points were calculated. Values less than or equal to the first tercile were defined as “low growth”, those greater than the first tercile but less than the second tercile were “medium growth,” and all remaining values were “high growth”. Additionally, Venn diagrams [130] were constructed to illustrate shared and unique substrate utilization phenotypes among the four-species consortium. For the Venn diagrams, the data was first split into two categories, “growth” and “no growth”. Data equal to or less than the lowest percentile were defined as “no growth”, whereas the remaining data were categorized as “growth”.

### 4.5 Clearing zones of different antibiotics

Susceptibility to various antibiotics was assessed on a solid surface as clearing zones surrounding Oxoid™ discs. Four different antibiotics representing three different classes (aminoglycosides, chloramphenicol, tetracyclines) were tested. 100 μL of overnight culture was mixed with 4 mL of top agar (0.4 % (W/V) agar, 1.5 % (W/V) TSB). This mixture was then tempered to 46 °C and plated on top of a TSA plate. After cooling for 5 min, sterile tweezers were used to place one of each antibiotic disc, (amoxicillin 25 μg, amoxicillin/clavulanic acid 30 μg, piperacillin 100 μg, piperacillin/tazobactam 110 μg, chloramphenicol 30 μg, gentamycin 30 μg, streptomycin 300 μg and tetracycline 10 μg) on the plates. Four discs were distributed per plate. Plates were incubated at 24 °C for 24 h before the clearing zones were measured.

### 4.6 Minimum inhibitory concentration (MIC) assessment

For the minimum inhibitory concentration (MIC) analysis, overnight cultures of *S. rhizophila* and *S. maltophilia* were diluted 100-fold, grown to exponential phase, and adjusted to OD_600_ = 0.1. The cultures were mixed with ampicillin concentrations of 800, 400, 200, 100, 50, 25, and 6.25 μg/mL. OD_600_ was measured over 20 h at 24 °C with continuous shaking in the microplate reader. A positive control with no antibiotics and a negative control with only TSB were included.

### 4.7 Azurine-cross linked polysaccharide (AZCL) assay for enzymatic capacities

AZCL-linked substrates (amylose, casein, curdlan, cellulose, xylan, galactomannan and rhamnogalacturonan I) were purchased from Megazyme. To prepare the AZCL mixture, 0.3 g of the appropriate AZCL substrate was mixed with 6 mL of EtOh and put on a RS-24 mini rotator (BIOSAN) for a minimum of 1 h. After thorough mixing, 45 mL of 0.1x TSA was combined with 1 mL of the AZCL + EtOH mixture. 23 mL was distributed per Petri dish. 200 μL pipette tips were used to puncture four holes in the agar, which were then used for the bacterial inoculant. To make the holes, a pipette tip was picked up at the narrow end by flamed-sterilized tweezers. The wide opening of the tip was then pressed into the agar plate, creating a hole. To test for enzymatic activity using these AZCL plates, bacterial overnight cultures were cultured as described above and diluted 10-fold. If necessary, the dilutions were mixed in the appropriate dual-, triple- and four-species combinations. 10 μL of each sample was inoculated into the punctured holes. The plates were then incubated for 24 h at 24 °C. Following incubation, the clearing zones of the azurine (blue) dye were measured and quantified for each AZCL substrate.

### 4.8 Genomic differentiation of *S. rhizophila* and *S. maltophilia*

*S. rhizophila* and *S. maltophilia* were compared and the unique genes in each were identified. First, a blast comparison file was generated comparing the two strains to each other. Then, a threshold of 70 % for the identity was set to filter the results. Next, the ‘comm’ command was used to generate an output of genes unique to one strain and absent in the other. This output was then input into the KAAS – KEGG Automatic Annotation Server [131]. Using the BHH job request, the GHOSTZ search program grouped the unique genes in both isolates relative to their KEGG pathways identification. The comparison files were also inputted into EggNOG-Mapper [127] to obtain the top unique gene functions for each *Stenotrophomonas* species.

The Comparative Systems tool from BV-BRC was used to compare the protein subsystems between these two genomes. Assessment of similarity between *S. rhizophila* and *S. maltophilia* genomes was computed with whole-genome Average Nucleotide Identity (ANI) (v. 1.33) [132,133] and synteny was analyzed with SynMap from CoGe [134].

## CRediT authorship contribution statement

**Dana Ronin:** Writing – review & editing, Writing – original draft, Visualization, Validation, Software, Methodology, Investigation, Formal analysis, Conceptualization. **Mads Frederik Hansen:** Writing – review & editing, Writing – original draft, Visualization, Validation, Methodology, Investigation, Conceptualization. **Maximilian Lukas Flaig:** Writing – review & editing, Visualization, Validation, Investigation. **Morten Kam Dahl Dueholm:** Writing – review & editing, Resources, Formal analysis, Data curation. **Anders Ogechi Hostrup Daugberg:** Resources, Formal analysis, Data curation. **Joseph Nesme:** Writing – review & editing, Resources, Data curation. **Witold Kot:** Writing – review & editing, Resources, Data curation. **Mette Burmølle:** Writing – review & editing, Supervision, Resources, Project administration, Methodology, Funding acquisition, Conceptualization.

## Declaration of competing interest

The authors declare the following financial interests/personal relationships which may be considered as potential competing interests: Mette Burmolle reports financial support was provided by 10.13039/501100000781European Research Council. If there are other authors, they declare that they have no known competing financial interests or personal relationships that could have appeared to influence the work reported in this paper.

## Data Availability

Data will be made available on request.
