# Supplementary material for: Unfolding the collective functional potential of a synergistic multispecies community through genotypic and phenotypic analyses
Source: Biofilm. 2025 May 24;10:100290. doi: 10.1016/j.bioflm.2025.100290 (PMC12246864; doi:10.1016/j.bioflm.2025.100290)
Supplement: Multimedia component 5 [file mmc5.docx]

**Supplementary Figures:**

**

***Supplementary figure 1****. The Enterococcus faecalis PS operon was identified in both Paenibacillus amylolyticus and Stenotrophomonas rhizophila. When the synteny of the identified PS operons was compared to the original E. faecalis query, marked differences were seen, with critical genes missing in our strains regarding product modification and polymerization.*

**A)**

**B)**

**C)**

***Supplementary figure 2****. Full BIOLOG data overview.* ***A)*** *GenIII BIOLOG plate.* ***B)*** *PM1 BIOLOG plate.* ***C)*** *PM2 BIOLOG plate.*

******

***Supplementary figure 3. Comparing the Stenotrophomonas spp. showed further distinctions between the two.*** *When comparing the similarity of the two Stenotrophomonas isolates of this community, Stenotrophomonas rhizphila and Stenotrophomonas maltophilia, we got an average nucleotide identity (ANI) estimate of 84,7%. Despite the high percentage of genomic similarity, the range of phenotypic differences between the two strains (i.e. biofilm formation, resistance profiles) prompted for a closer genotypic comparison.* ***A)*** *Genome synteny graph between Stenotrophomonas rhizophila and Stenotrophomonas maltophilia. The break in the center of the synteny plot suggests either unique genes present or a variation in the order of genomic features between the two strains in that relative genomic location.* ***B)*** *Comparison between Stenotrophomonas rhizophila and Stenotrophomonas maltophilia was done to identify unique genes in each of them. They differ by 81 genes (45 in Stenotrophomonas maltophilia and 36 in Stenotrophomonas rhizophila) in two-component systems, and 27 genes (6 in Stenotrophomonas maltophilia and 21 in Stenotrophomonas rhizophila) in flagella assembly.* *There are also 6 genes that are unique for glycerophospholipid metabolism in Stenotrophomonas maltophilia, to which Stenotrophomonas rhizophila has no alternatives for. The analysis for these unique genes were then categorized by KEGG pathways, shown above. A cutoff of >5 gene hits was set per pathway (pathways less than 5 were included in the case one of the strains had >5 hits for that pathway).* ***C+D)*** *Growth curve for Stenotrophomonas maltophilia and Stenotrophomonas rhizophila, respectively, on increasing concentrations of ampicillin (µg/mL). While both species were able to grow at the maximum concentration tested (800µg/mL), Stenotrophomonas rhizophila exhibited a dose-dependent growth delay while Stenotrophomonas malthophilia grew unhindered in increasing concentrations of ampicillin.*

**

***Supplementary figure 4.*** *Standard curve for OD to CFU/ml conversion.*

**Supplementary tables**

***Supplementary table 1.*** *The superfamily of proteins of S-Ribosylhomocysteinase (LuxS) (pfam entry PF02664) were blasted against the four strains. Protein matches between the strains and luxS proteins were then filtered to include matches with identity of 85% or above.*

| Strain | Locus ID | Product | Match to pfam ID | % Match |
| --- | --- | --- | --- | --- |
| *P. amylolyticus* | PWP87_00960 | Extracellular solute-binding protein | A0A1X6X8A6\|unreviewed\|S-ribosylhomocysteine | 90.91 |
| *P. amylolyticus* | PWP87_01870 | Class I SAM-dependent methyltransferase | A0A7W8HB43\|unreviewed\|S-ribosylhomocysteine | 100.00 |
| *P. amylolyticus* | PWP87_16445 | Barstar family protein | A0A2N5M4K2\|unreviewed\|S-ribosylhomocysteine | 90.00 |
| *M. oxydans* | PWP88_02995 | MoaD/ThiS family protein | A0A9D2AJJ2\|unreviewed\|S-ribosylhomocysteine | 90.00 |
| *S. rhizophila* | PWP89_01110 | Hypothetical protein | J1GX31\|unreviewed\|S-ribosylhomocysteine | 100.00 |
| *S. maltophilia* | PWP90_04045 | Hypothetical protein | A0A660NVG6\|unreviewed\|S-ribosylhomocysteine | 90.00 |

***Supplementary table 2.*** *Here is the full list of protein hits which matched the NCBI database for ‘biofilm matrix’ identical protein group matches. The protein matches between the strains and the database were then filtered to only include matches with a percent identity of 70% or above. The best hit to the protein in the database is also shown.*

***Supplementary table 3.*** *List of genes in each respective secondary metabolite gene cluster identified with the antiSMASH standalone database. The four sheets are labelled accordingly for the genes identified in Paenibacillus amylolyticus, Microbacterium oxydans, Stenotrophomonas rhizophila, and Stenotrophomonas maltophilia.*

***Supplementary table 4.*** *The full list of antimicrobial resistant genes for the 4 strains.*

| AMR Mechanism | *P. amylolyticus* | *M. oxydans* | *S. rhizophila* | *S. maltophilia* |
| --- | --- | --- | --- | --- |
| Antibiotic activation enzyme | - | - | KatG | KatG |
| Antibiotic inactivation enzyme | CatA15/A16 family, FosB | - | - | APH(3')-II/APH(3')-XV, L1 family |
| Antibiotic target protection protein | - | - | - | QnrB family |
| Antibiotic target in susceptible species | Alr, Ddl, dxr, EF-G, EF-Tu, folA, Dfr, folP, gyrA, gyrB, Iso-tRNA, kasA, MurA, rho, rpoB, rpoC, S10p, S12p | Alr, Ddl, dxr, EF-G, EF-Tu, folA, Dfr, folP, gyrA, gyrB, Iso-tRNA, kasA, MurA, rho, rpoB, rpoC, S10p, S12p | Alr, Ddl, dxr, EF-G, EF-Tu, folA, Dfr, folP, gyrA, gyrB, Iso-tRNA, kasA, MurA, rho, rpoB, rpoC, S10p, S12p | Alr, Ddl, dxr, EF-G, EF-Tu, folA, Dfr, folP, gyrA, gyrB, Iso-tRNA, kasA, MurA, rho, rpoB, rpoC, S10p, S12p |
| Antibiotic target modifying enzyme | RlmA(II) | Erm(X) | - | - |
| Antibiotic target replacement protein | fabV | FabL-like | fabV | fabV |
| Efflux pump conferring antibiotic resistance | BceA, BceB, MacA, YkkCD | Tet(42) | EmrAB-OMF, EmrAB-TolC, MacA, MacB, MdtABC-TolC, TolC/OpmH | EmrAB-OMF, EmrAB-TolC, MacA, MacB, MdtABC-TolC, TolC/OpmH |
| Gene conferring resistance via absence | gidB | gidB | gidB | gidB |
| Protein altering cell wall charge conferring antibiotic resistance | GdpD, PgsA | GdpD, PgsA | PgsA | PgsA |
| Protein altering cell wall structure conferring antibiotic resistance | VanXY-unclassified | - | - | - |
| Regulator modulating expression of antibiotic resistance genes | BceR, BceS, LiaR, LiaS, VanF/M-type | MtrA, MtrB, VanO-type | OxyR | OxyR |

***Supplementary table 5***. *Social genes identified by SOCfinder were then categorized into KEGG pathways.*

***Supplementary table 6.*** *The compiled list of the metabolic pathways identified in Stenotrophomonas rhizophila and Stenotrophomonas maltophilia, with a specific focus on differential pathways identified (shown in yellow). The Comparative Systems tool from BV-BRC was used for metabolic pathways identification.*
